# Data on modeling of enzymatic elimination of Direct Red 81 using Response Surface Methodology

**DOI:** 10.1016/j.dib.2018.03.012

**Published:** 2018-03-08

**Authors:** Hossein Kamani, Gholam Hossein Safari, Ghorban Asgari, Seyed Davoud Ashrafi

**Affiliations:** a Health Promotion Research Center, Zahedan University of Medical Sciences, Zahedan, Iran; b Health and Environmental Research Center, Department of Environmental Health, School of Public Health, Tabriz University of Medical Sciences, Tabriz, Iran; c Social Determinants of Health Research Center (SDHRC), Department of Environmental Health Engineering, Hamadan University of Medical Sciences, Hamadan, Iran; d School of Health, Guilan University of Medical Sciences, Rasht, Iran; e Research Center of Health and Environment, Guilan University of Medical Sciences, Rasht, Iran

**Keywords:** Laccase, Dye, Direct Red 81, Elimination, Box–Behnken

## Abstract

In this article, three variables including laccase dose, 2,2'-Azinobis-(3-ethylbenzothiazoline-6-sulfonate) (ABTS) dose and pH were used to modeling of Direct Red 81 (DR81) elimination from aqueous solutions by laccase-mediated system. Obtained data indicated that the predicted and experimental values were close for DR81 elimination, and the regression was also able to give a good prediction of response for DR81 elimination (*R*-Squared = 0.9983). From the experimental, the highest elimination of the DR81 was 95. 5% after 30 min incubation at pH 5, temperature 40 °C, ABTS 0.2 mM, and initial concentration of DR81 50 mg L^−1^ in the presence of 0.2 U mL^−1^ of the laccase. The data showed that the laccase can be used as a "green" technology for treating of dyes from aqueous solutions. Data analysis was performed using Design-Expert version 7.0.0 (Stat-Ease, trial version).

## Specifications Table

**Table:** 

Subject area	*Environmental Sciences*
More specific subject area	*Biotechnology*
Type of data	*Figure and table*
How data was acquired	*The study was started by adding laccase to the reaction solutions (final volume of 5 mL). Samples were taken after incubation time (30 min).The residual concentrations of DR81 were done through a calibration curve by reading the maximum absorbance wavelength 509 for DR81, using UV–vis spectrophotometer (Shimadzu UV 1700, Japan). Digital pH meter (Metrohm) was applied for pH analyzing*.
Data format	*Raw, analyzed*
Experimental factors	*The main and interaction influence of solution pH, laccase activity and ABTS concentration was evaluated during the experiments of DR81 elimination.*
Experimental features	*DR81 elimination by enzymatic process was done and its efficiency was determined*.
Data source location	*Department of Environmental Health Engineering, School of Health, Guilan University of Medical Sciences, Rasht, Iran*.
Data accessibility	*All data are available within paper*.

## Value of the data

• The data of laccase-mediated process was described for DR81 elimination from aqueous solution.
• The data will be useful for application of laccase for treatment of industrial wastewater including DR81 and similar synthetic dyes. Enzymatic elimination of pollutants from the environment is one of the interesting methods that known as a "green" technology.
• This data will be useful to the researchers and scientific community wanting to analyze the ability of laccase for DR81 elimination from aqueous solution.

## 1 Data

**Fig. 1:**
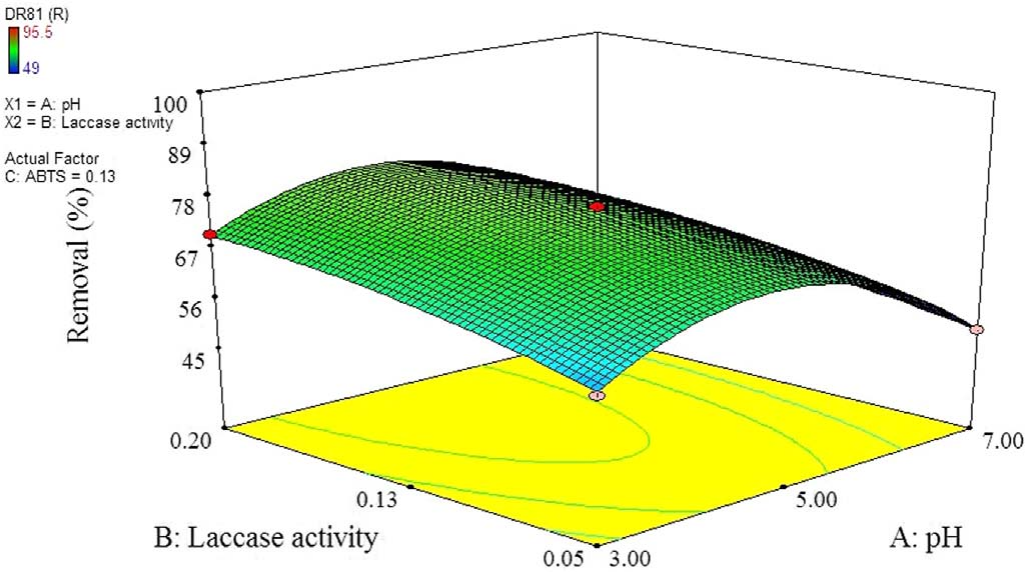
3D surface plot from BBD showing the interaction effects of pH and laccase activity on elimination of DR81.

**Fig. 2:**
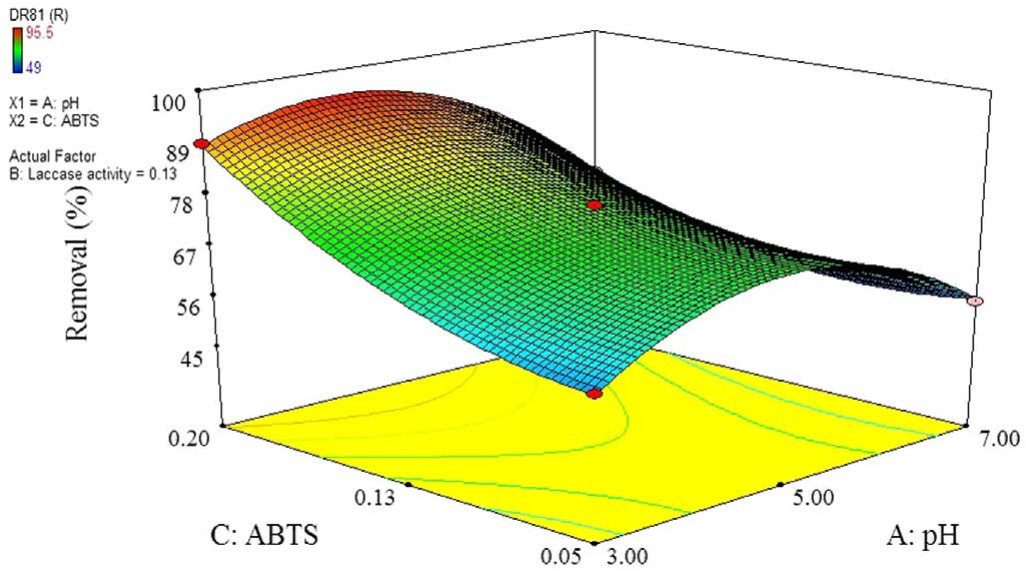
3D surface plot from BBD showing the interaction effects of pH and ABTS on elimination of DR81.

**Fig. 3:**
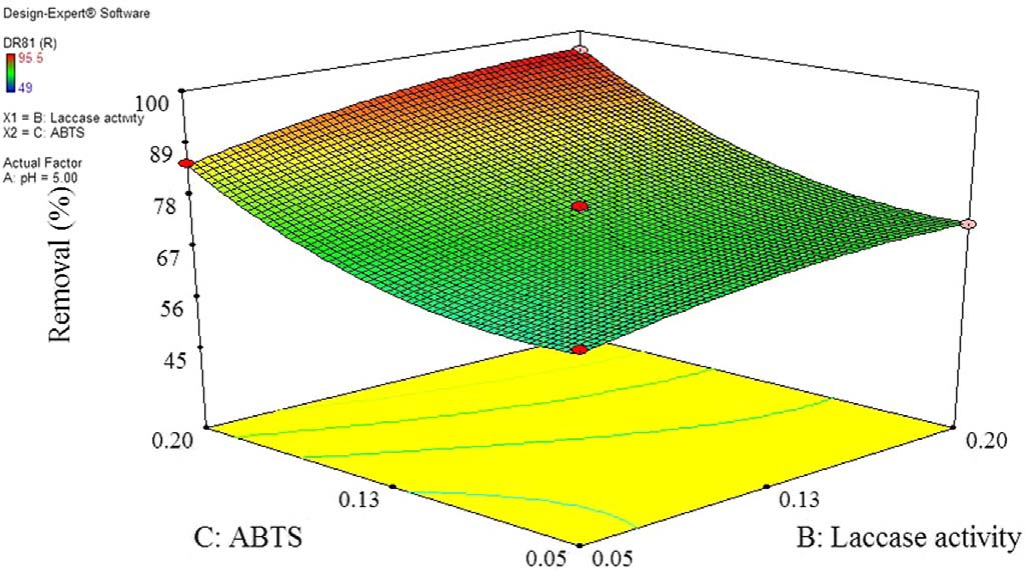
3D surface plot from BBD showing the interaction effects of ABTS and laccase activity on elimination of DR81.

**Fig. 4:**
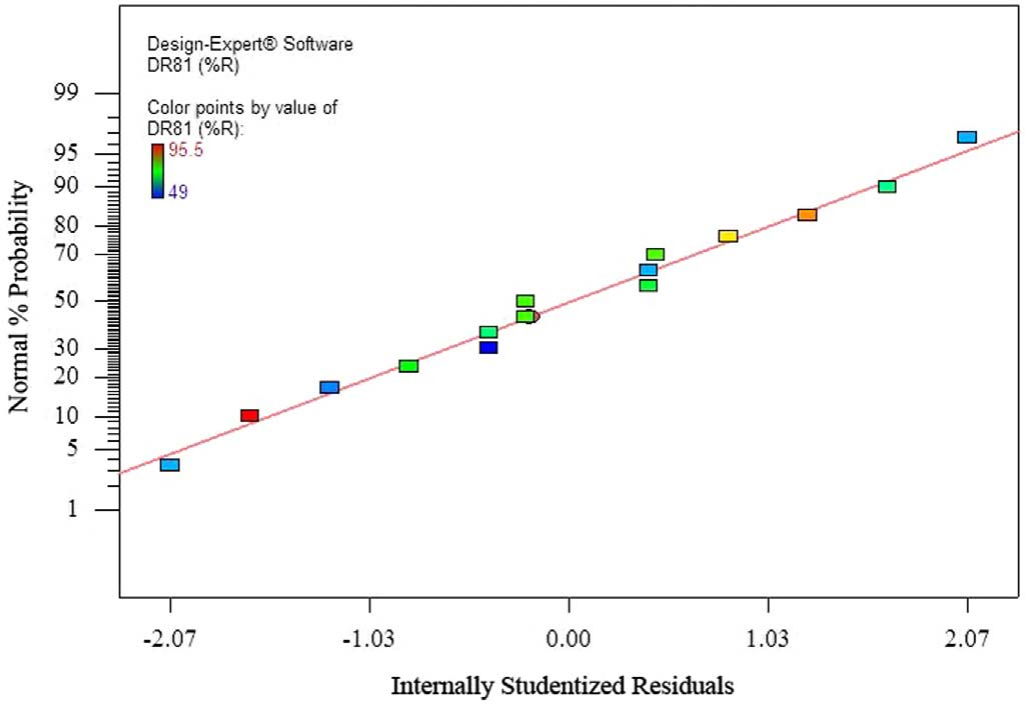
Normal probability plots of internally studentized residuals for DR81 elimination.

**Fig. 5:**
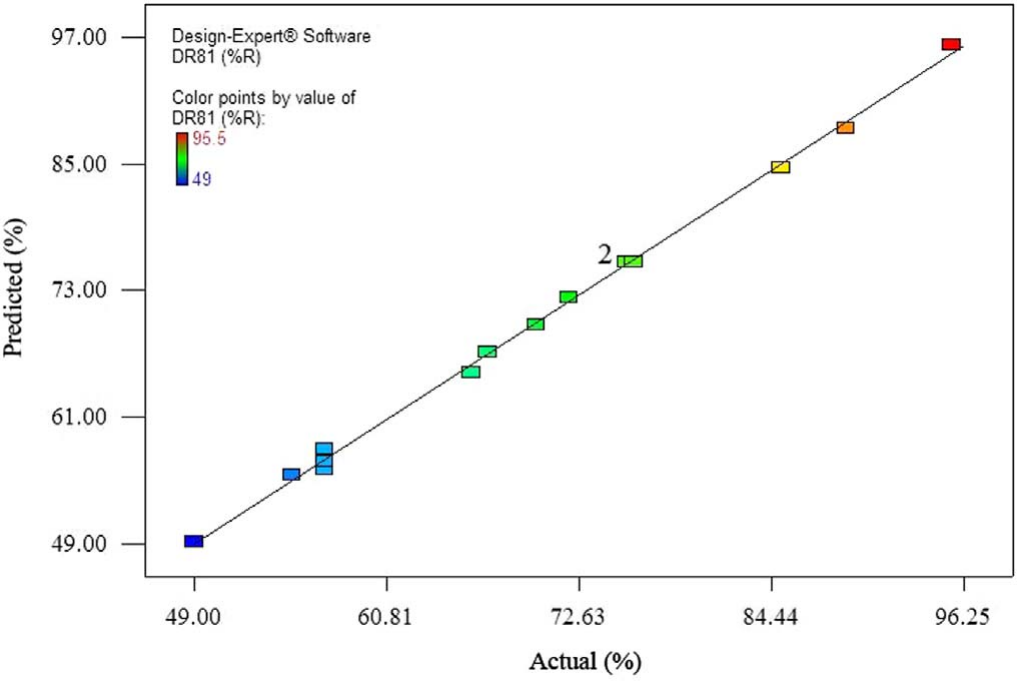
Comparison of experimental data with the RSM model predictions.

**Fig. 6:**
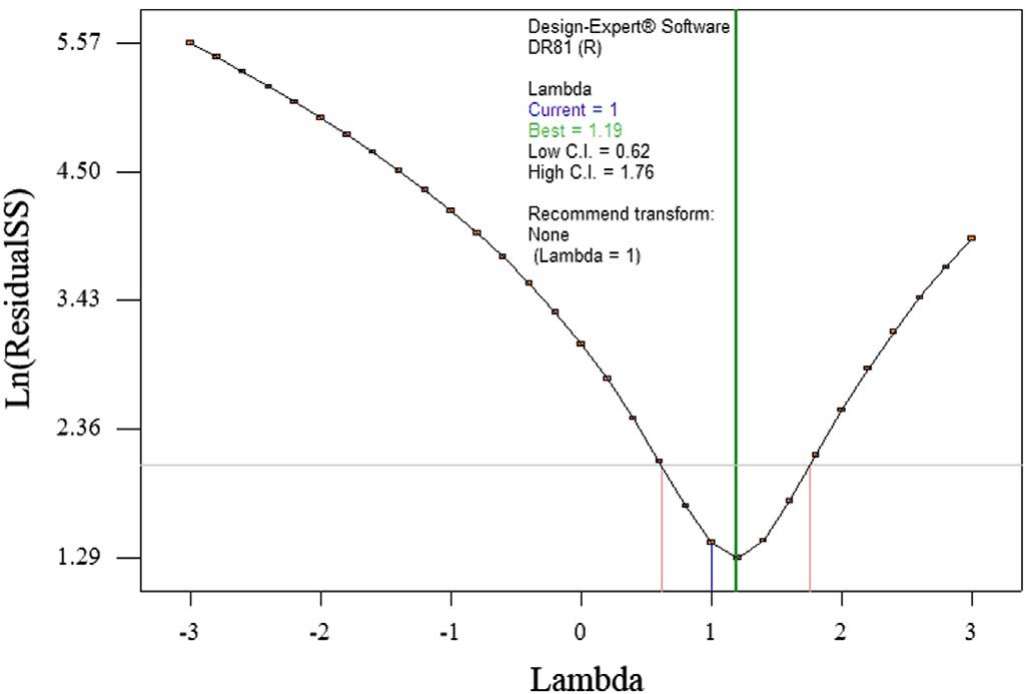
Box-Cox plot of RSM model transformation.

The data of this paper showed the elimination of DR81 dye using the laccase-mediated system. Data in Table 1 gives information about general characteristic of DR81 dye. The three studied variables (pH, laccase dose and ABTS dose) and their levels have been shown in Table 2. The experimental design by Box–Behnken Design (BBD), actual, predicted, and residual values of DR81 elimination efficiency have been provided in Table 3. According to the obtained data from experiments, the maximum elimination of DR81 was 95.5%, whereas its predicted value was 96.25% from model indicating a good agreement between experiment and model. According to the *p*-value of main and interaction effects of all three studied variables that obtained by Analysis of Variance, ANOVA, (Table 4), the main and interaction effects of laccase dose, ABTS dose and pH on DR81 elimination process were statically significant (*p*-value < 0.05). The interaction effects of studied variables on the DR81 elimination efficiency have been shown in Fig. 1, Fig. 2, Fig. 3. The normal probability plot of the residuals and the parity plot comparing the elimination efficiency of the experimental vs model predicted have been shown in Fig. 4, Fig. 5, respectively. The Box–Cox plot of a natural log (Ln) of the residual sum of square vs lambda has been shown in Fig. 6. By ANOVA, the quadratic equation for DR81 elimination using pH (*A*), laccase dose (*B*), and ABTS dose (*C*) as the main variables is as Eq. (1);

(1) $$(\%)\;\text{R}\;=+\mathrm{75}\mathrm{.67}\;-\;5.62A\;+\;4.69B\;+\;\mathrm{10}.81C\;-\;1.25\mathrm{AB}\;-\;5\mathrm{AC}\;-\;\mathrm{15}.02A^{2}\;-\;2.4B^{2}\;+\;6.35C^{2}$$

**Table 1:** General characteristic of DR81 [4], [9].

**Parameter**	**Characteristic**
Chemical name	Direct Red 81
C.I. number	28,160
Classification	Diazo
Apparent colour	Red
Molecular weight	675.59
Molecular formula	C_29_H_19_N_5_Na_2_O_8_S_2_
*λ*_*max*_ (nm)	509
Chemical structure

**Table 2:** Three studied variables and levels.

**Independent variables**	**Unit**	**Factors**	**Actual and coded values**		
			**− 1**	**0**	**1**
pH	–	A	3	5	7
Laccase dose	U mL^−1^	B	0.05	0.125	0.2
ABTS dose	mM	C	0.05	0.125	0.2

**Table 3:** BBD matrix of variables along with observed, predicted, and residual values.

**Run**	**Levels**			**Response**		
	***A***	***B***	***C***	**Observed**	**Predicted**	**Residuals**
1	3	0.05	0.125	57	57.937	− 0.937
2	7	0.05	0.125	49	49.187	− 0.187
3	3	0.2	0.125	70	69.812	0.187
4	7	0.2	0.125	57	56.062	0.937
5	3	0.125	0.05	57	56.812	0.187
6	7	0.125	0.05	55	55.562	− 0.562
7	3	0.125	0.2	89	88.437	0.562
8	7	0.125	0.2	67	67.187	− 0.187
9	5	0.05	0.05	66	65.250	0.750
10	5	0.2	0.05	72	72.375	− 0.375
11	5	0.05	0.2	85	84.625	0.375
12	5	0.2	0.2	95.5	96.250	− 0.750
13	5	0.125	0.125	75.5	75.666	− 0.166
14	5	0.125	0.125	75.5	75.666	− 0.166
15	5	0.125	0.125	76	75.666	0.333

**Table 4:** ANOVA for the fitted quadratic model of DR81 elimination.

**Source**	**Degrees of freedom**	**Sum of squares**	**Mean square**	***F*-value**	***p*-value**	**Status**
**Model**	9	2529.8	281.0	342.4	< 0.0001	Significant
***A***	1	253.1	253.1	308.3	< 0.0001	Significant
***B***	1	175.7	175.7	214.1	< 0.0001	Significant
***C***	1	935.2	935.2	1139.4	< 0.0001	Significant
***AB***	1	6.2	6.2	7.6	0.0399	Significant
***AC***	1	100	100	121.8	0.0001	Significant
***BC***	1	5.0	5.0	6.1	0.0556	Significant
***A***^**2**^	1	833.0	833.0	1014.9	< 0.0001	Significant
***B***^**2**^	1	21.1	21.1	25.8	0.0038	Significant
***C***^**2**^	1	149.0	149.0	181.6	< 0.0001	Significant
**Residual**	5	4.1	0.8			
**Lack of Fit**	3	3.9	1.3	15.7	0.0603	Insignificant
**Pure Error**	2	0.1	0.0			
**Cor Total**	14	2533.9				

*R*-Squared = 0.0.9983, Adjusted *R*-Squared = 0.9954, Adequate Precision = 63.6.

## 2 Experimental design, materials and methods

### 2.1 Materials

Laccase (EC 1.10.3.2, p-benzenediol:dioxygen oxidoreductases) from *Trametes Versicolor* (activity > 10 U mg^−1^) [1], [2], and ABTS, were purchased from Sigma Aldrich (St. Louis, MO, USA). The synthetic dye (DR81) was obtained from Alvan Sabet Co. (Tehran, Iran). All other chemicals were of the highest purity available.

### 2.2 Analytical measurements

#### 2.2.1 Laccase assay

As described by Rekuć et al. [1], [2], [3], monitoring the oxidation of 1 mL of 2 mM ABTS as a substrate (using UV–Vis spectrophotometer, *λ_max_* 420 nm) in a reaction mixture containing 0.1 M sodium citrate buffer (pH 4.5) and 1 mL of diluted enzyme sample at 40 °C, the laccase activity was calculated. One activity unit was defined as the amount of enzyme that oxidized 1 μmol of ABTS per min [1], [3].

#### 2.2.2 Determination of dye concentration

The analysis of dye concentration was done through a calibration curve by reading the maximum absorbance wavelength 509 nm for DR81, using UV–vis spectrophotometer. The removal percentage was then determined by the following equation (Eq. (2));

(2) $$\text{Removal}\;(\%)=100\times \frac{C_{0}\;-\;C_{t}}{C_{0}}$$

where *C_t_* is the concentration (mg L^−1^) at the end of process time and *C_0_* is the initial concentration (mg L^−1^) of dye [4], [5].

### 2.3 Experimental design

#### 2.3.1 BBD by Response Surface Methodology (RSM)

RSM can be used as a statistical tool to determine the main and interaction effects of variables [6], [7], [8]. In order to evaluate the effect of three variables (pH, laccase dose and ABTS dose) on DR81 elimination efficiency and elimination optimum conditions, an experimental design using BBD was used by using Design-expert version 7.0.0 (Stat-Ease, trial version) software. The results of the experimental design were analyzed and along with the main effects, interactions and quadratic effects of all variables were determined.

#### 2.3.2 DR81 elimination experiments

DR81 (50 mg L^−^^1^) solution was prepared in citrate sodium buffer (0.1 M). The elimination studies were started by adding laccase according to the pH and ABTS (Table 3), solution volume of 5 mL, temperature 40 °C, and 150 rpm under dark for 30 min [2], [3].

## Figures and Tables

**Figure:**
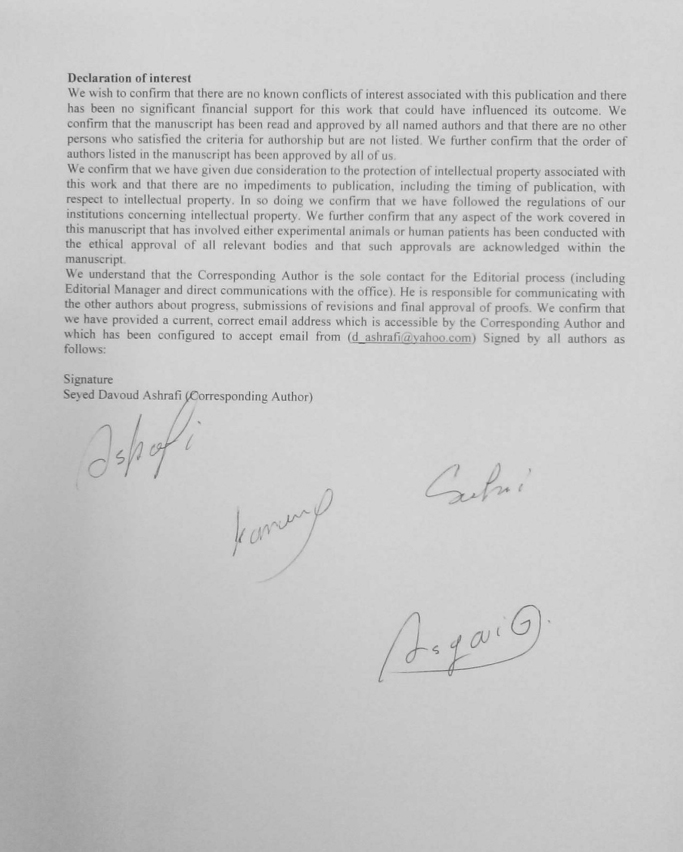
Fig. S1.
